# Functional coordination of muscles underlying changes in behavioural dynamics

**DOI:** 10.1038/srep27759

**Published:** 2016-06-10

**Authors:** Carlijn A. Vernooij, Guillaume Rao, Dionysios Perdikis, Raoul Huys, Viktor K. Jirsa, Jean-Jacques Temprado

**Affiliations:** 1 Aix-Marseille Université, CNRS, Institut des Sciences du Mouvement UMR 7287, 13288, 13009, Marseille, France; 2 Aix Marseille Université, Inserm, Institut de Neurosciences des Systèmes UMR_S 1106, 13005, Marseille, France; 3 Max Planck Institute for Human Development, Center for Lifespan Psychology, Berlin, Germany

## Abstract

The dynamical systems approach addresses Bernstein’s degrees of freedom problem by assuming that the neuro-musculo-skeletal system transiently assembles and dismantles its components into functional units (or synergies) to meet task demands. Strikingly, little is known from a dynamical point of view about the functioning of the muscular sub-system in this process. To investigate the interaction between the dynamical organisation at muscular and behavioural levels, we searched for specific signatures of a phase transition in muscular coordination when a transition is displayed at the behavioural level. Our results provide evidence that, during Fitts’ task when behaviour switches to a different dynamical regime, muscular activation displays typical signatures of a phase transition; a reorganisation in muscular coordination patterns accompanied by a peak in the variability of muscle activation. This suggests that consistent changes occur in coordination processes across the different levels of description (i.e., behaviour and muscles). Specifically, in Fitts’ task, target size acts as a control parameter that induces a destabilisation and a reorganisation of coordination patterns at different levels of the neuro-musculo-skeletal system.

The hypothesis that movement production relies on the emergence of coordinative structures in which the individual components (e.g. neurons, muscles, joints, limbs) are grouped together as functional units^1^ has been widely echoed in the neuroscience community, notably by the proponents of the dynamical systems approach^2^,^3^. In this perspective, it is considered that the human neuro-musculo-skeletal system (NMSS) has the capacity of transiently assembling and dismantling its components into functional units (or synergies, modes, etc.) to (self-) organise into regimes to meet different task demands^2^,^3^. As an illustration, this approach allowed identifying pattern formation, at behavioural level, in a wide number of task paradigms, including Fitts’ task e.g.^4^,^5^,^6^.

In Fitts’ task, participants are instructed to move as fast as possible between two targets with width W and inter-target distance D^7^. Varying W and/or D changes task difficulty, which can be quantified by the index of difficulty^8^


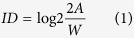


When increasing task ID by decreasing W, a phase transition is observed at the behavioural level^5^: the dynamical regime changes from a limit cycle (expressed by continuous movements) to fixed points (expressed by discrete movements per half-cycle). This phase transition has been demonstrated by phase flow analyses^4^,^5^, which have indicated the presence of fixed points at higher IDs only (see^9^ for a different method to identify phase transitions in Fitts’ task). Higher IDs are also associated with a modified endpoint kinematic pattern, as revealed by a decreased acceleration-to-deceleration ratio^6^. A phase transition in behavioural dynamics is a key signature of different control mechanisms which is assumed to express, at behavioural level, the coordination across multiple elements in the NMSS, including muscles.

However, while there has been much debate about whether the muscular subsystem matters for coordination dynamics, the question of *how* the muscles are coordinated has received only limited attention from the dynamical system perspective (see^10^,^11^ for notable exceptions). This is surprising as so far it is not clear whether and how muscular coordination patterns change during behavioural phase transitions. Neither is it known whether, depending on the type of processes that mediate the coupling between muscles, any coordination pattern at the muscular level can be identified through the analysis of electromyography (EMG: measure of the ‘neural input command to the muscles’). Together with the unquestionably complex and non-linear link between muscle activations and muscle forces^12^,^13^,^14^,^15^, the presence of muscular redundancy (i.e., more muscles than degrees of freedom at the joints) leads to an impossibility to directly relate EMG to endpoint kinematics. Thus, it is not obvious whether any dynamical (re)organisation of muscles can be captured by EMG or for instance by recording muscle force (the ‘mechanical output’). As it is currently still impossible to measure *in vivo* individual muscle forces (though see e.g.^16^ for advancements in estimations), EMG recordings will be the sole candidate examined in the present study. To our knowledge, only two studies^17^,^18^ have measured EMG in Fitts’ task. Whereas they mainly focussed on co-contraction, their results indicated a potential for reorganisation of muscular coordination with increasing accuracy constraints.

Here, we study how the muscular system behaves during a transition between qualitatively distinct dynamical regimes in behaviour observed in Fitts’ task. Our general assumption was that a behavioural phase transition between dynamical regimes expresses underlying coordination processes within and between the different levels of the whole NMSS. Accordingly, our approach consists of identifying the general phenomena of coordination and revealing their underlying implementation level by level, that is, in the present study, at behavioural and muscular levels. In the framework of coordination dynamics, typical signatures accompany a phase transition^2^,^3^, namely: i) a reorganisation of the system from one low dimensional coordinative pattern to another, ii) critical fluctuations expressed as a marked increase in variability of the system’s behaviour before transition, which is indicative of a loss of stability of the current coordination pattern, and a decrease in variability after the transition, indicating a higher stability of coordination after switching to the new pattern. Here, we study whether these typical signatures are displayed at muscular level when a phase transition between different dynamical regimes in behaviour occur during Fitts’ task^19^,^20^,^21^.

To capture a reorganisation in muscular coordinative patterns and critical fluctuations, we implemented and adopted methods from fields outside of the dynamical systems approach. There are several techniques to extract muscular coordinative patterns, often called ‘muscle synergies’ (e.g. non-negative matrix factorization, independent component analysis, factor analysis), which all have specific underlying assumptions and constraints. Nevertheless, they have been shown to lead to similar results for the weighting and the temporal components of the muscular coordinative patterns^22^. To enable a detailed view of the reorganisation process we adopted a similar approach as De Marchis *et al*.^23^, where non-negative coordination patterns are extracted per small time-bin of EMG data which are clustered together thereafter. In this context, we define a synergy in line with the dynamical systems approach^24^ as a temporal functional coordination pattern that can evolve during the realization of the task. To measure critical fluctuations, we used functional connectivity dynamics (FCD) analysis, which was previously utilised for fMRI data^25^. This method was developed to address the unsatisfying assumption that the functional connectivity (FC) in the brain (i.e., among BOLD activations at different brain areas) is constant throughout a multiple minutes recording session^26^. Here, we applied it to EMG data to study whether muscular activations show a peak in their coordination variability in parallel to a phase transition in behaviour. Such increase in variability would be considered as a typical signature (among other signs) of a phase transition at the muscular level.

We tested the following predictions. First, we expected to observe an increase in FCD variability in muscular coordination at the vicinity of the phase transition between behavioural dynamical regimes and a lower FCD variability of muscular coordination both before and after the behavioural transition, revealed by the phase flow analysis. Second, we expected to observe a different coordination of muscular activation patterns associated with the different behavioural dynamical regimes. This could express itself as either a change in the number of the synergies and/or a change in the nature of such synergy patterns. Alternatively, we could expect any reorganisation during Fitts’ task to express itself as an alternate activation of a fixed repertoire of synergies, as comparable movements with different task constraints as well as transitions in gait have shown to be produced by an alternate organisation of a set repertoire of synergies^27^,^28^,^29^,^30^,^31^.

## Results

### Identification of change in behavioural dynamical regimes

**Figure 1:**
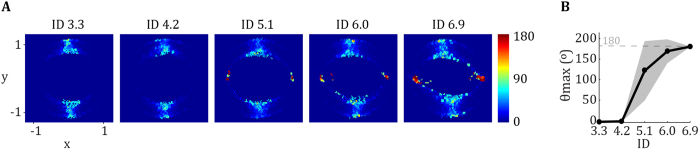
(**A**) Reconstructed angle diagrams as a function of ID averaged across participants. The horizontal axes represent normalized position (*x*); the vertical axes normalized velocity (*y*). The colour coding (right side of the panel) represents the maximum angle in degrees between adjacent vectors. Red areas indicate the existence of locally opposing angles and imply the presence of a fixed point. Its absence implies the existence of a limit cycle. (**B**) The maximum angle in degrees between adjacent vectors as a function of ID averaged across participants (*θ*_max_). The horizontal axis represents ID; the vertical *θ*_max_ (degrees).

We reconstructed the dynamical vector fields for the behavioural profiles during our reciprocal, unimanual Fitts’ task (Fig. 1A), where the maximal angle between neighbouring vectors (*θ*_max_) indicates whether a fixed point (*θ*_max_ ~180°) or a limit cycle (*θ*_max _≪ 180°) controls the movement. When ID was low, *θ*_max_ was low also (<10°). With higher ID, *θ*_max_ showed a rather sharp transition to ~180°. The inflection point of the sigmoidal relationship of *θ*_max_ over ID was located at 4.9 ± 0.60 bits (Fig. 1B). The inflection point of the phase flows was presumably paralleled by changed kinematics. *MT*, *AT* and *DT* per half-cycle (~aiming movement) increased monotonously with ID (F_2.96,124.41_ = 3169.32, η^2^ = 0.99; F_4,168_ = 1906.51, η^2^ = 0.98; F_2.49,104.44_ = 2570.02, η^2^ = 0.98; respectively. All p < 0.001). Post hoc analyses showed that all IDs were significantly different from each other for all three variables. We found a breakpoint in the *AT*/*DT* ratio close to ID 5.1 (at ID = 5.14 bits) mirroring the inflection point in the phase flow. After this ID, the *AT*/*DT* ratio was decreased. See supplement for Figure S1 and further details on the kinematics.

### Quantification of variability through Functional Connectivity Dynamics analysis

**Figure 2:**
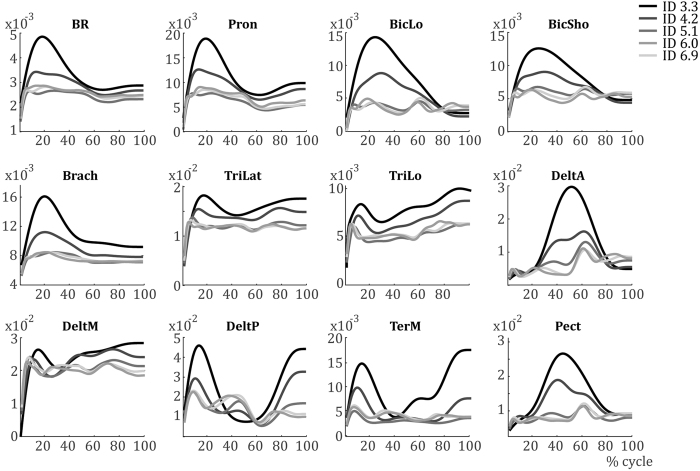
EMG activations per muscle averaged over all subjects for each condition.

The average muscular activation patterns per ID are shown in Fig. 2. There is a clear difference in EMG activity between IDs. Specifically, the activations are larger and smoother with lower IDs. For higher IDs, muscular activations are less pronounced in general. Additionally, some difference in the phasing of muscular activations can be noted. While unable to test it, a change in the shape of activation between IDs is shown in many muscles. For instance, DeltA shows an anticipatory activation for ID 3.3 to start the forward motion, which starts ~50% cycle, whereas its activation starts increasing not until after 50% cycle for ID 6.0 and ID 6.9.

**Figure 3:**
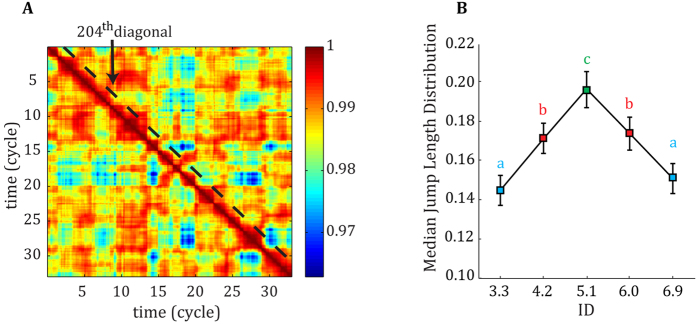
(**A**) Example of an FCD matrix. (**B**) Jump length distribution (*JLD*) curve of the FCD per ID across participants. P < 0.05. Error bars denote 1 standard deviation. Different letters above each error bar indicate significantly different groups.

Muscular activation of twelve upper limb and shoulder muscles were recorded and a measure of the variability in their coordination pattern was calculated (see Methods for a list of the recorded muscle). To study variability in muscular coordination patterns, we applied Functional Connectivity Dynamics (FCD) analyses to recorded EMG signals. The median jump length (*JLD*) of the FCD showed that there were larger fluctuations in correlational patterns among muscles at the ID where behaviour shows a transition between limit cycle and fixed point regimes (inverted U-shape modulation, see Fig. 3). At the transition, consistently larger jumps in muscular coordination, as quantified via pairwise correlations, were shown compared to away from the transition (F_2.96,162.94_ = 11.31, η^2^ = 0.17, p < 0.001). Post hoc analyses showed that the average *JLD* for ID 5.1 was significantly larger than for other IDs (all p < 0.001). Additionally, the average *JLD* for ID 4.2 and 6.0 were significantly larger than for ID 3.3 and 6.9 (all p < 0.001).

### Identification of muscle synergies

**Figure 4:**
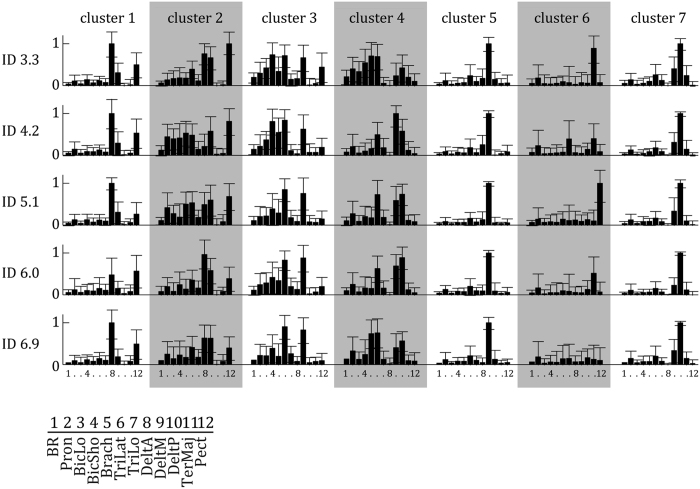
Synergy activation coefficients (+STD) extracted by NNMF as a function of ID averaged per cluster. Per synergy, the normalised activity of each of the twelve muscles is represented. Each muscle is activated in multiple synergies. Higher bars indicate increased strength of activity.

Two to seven muscle synergies (*W*) were extracted per two-cycle bin to account for a minimum of 90% of variance in the EMG data of the twelve muscles we recorded. With higher IDs, increasingly more *W* were extracted (mean ± std per bin: 3.12 ± 0.66, 3.43 ± 0.72, 3.60 ± 0.86, 3.72 ± 0.96, 3.85 ± 0.90, respectively, F_3.31,3237.48_ = 200,79, η^2^ = 0.17, p < 0.001, all post hoc pairwise p < 0.001). The cluster analysis on Sammon’s map values classified seven clusters per ID. Except for cluster 6, all *W*_*cluster*_, which represent the general activation pattern within a cluster, were comparable between IDs (Fig. 4, see Methods for abbreviations of muscles). Each *W*_*cluster*_ has a different relative activation of muscles, and a few strongly activated muscles i.e. DeltA and Pect in cluster 1; TriLat, DeltA, DeltM and Pect in cluster 2; BicSho, TriLat and DeltM in cluster 3; Pron, Brach, BicSho, TriLat, DeltM and DeltP in cluster 4; DeltM in cluster 5; Pron, TriLo, TerMaj and Pect in cluster 6; and DeltP in cluster 7. Most muscles are activated in multiple *W*_*cluster*_ and most *W*_*cluster*_ activate multiple muscles. Note that due to our extraction of synergies per 2-cycle bin and the subsequent clustering method, error-bars in Fig. 4 represent a combination of between-subject and within-subject variability. Although based on this information the consistency across participants cannot be estimated, this does give some indication of the variability in the synergies extracted in general.

**Figure 5:**
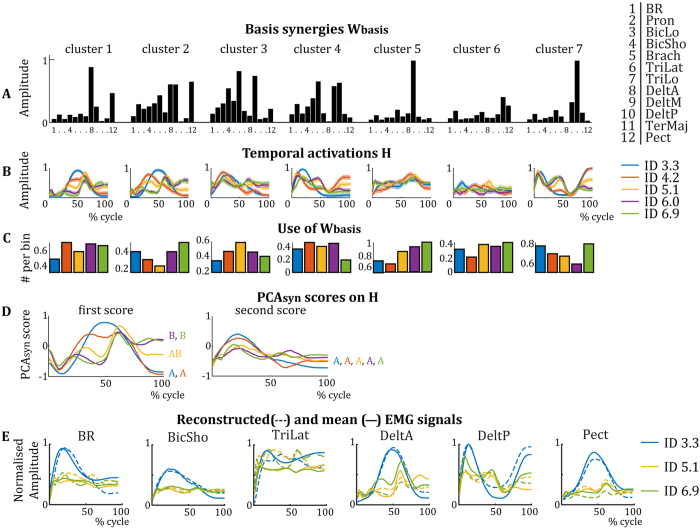
(**A**) Synergy activation coefficients from Fig. 4 averaged per cluster (*W*_*basis*_). Per basis synergy, the normalised activity of each of the twelve muscles is represented. Higher bars indicate increased strength of activity. (**B**) Average temporal activation components over a movement cycle per basis synergy per ID. Darker colours represent lower IDs. (**C**) Normalised frequency of use of the basis synergies per ID. (**D**) First and second *PCA*_*syn*_scores on the temporal activations *H* of panel B. The first *PCA*_*syn*_ score shows a clear difference between the activations of the IDs, activations with significant correlations (p < 0.001) are grouped by letters A and B, whereas the second *PCA*_*syn*_ score does not (all activations are correlated with all others). (**E**) Reconstructed EMG signals based on the synergies presented in ***A*** and ***B*** for ID 3.3, ID 5.1 and ID 6.9. The average EMG signals from Fig. 2 are superimposed for comparison.

As *W*_*cluster*_ were rather similar across IDs, they were averaged over ID to form basis synergies (*W*_*basis*_, Fig. 5). These *W*_*basis*_ (Fig. 5A) and their associated temporal activation profiles *H* (Fig. 5B) are able to reconstruct the average EMG signals presented in Fig. 2 rather well. In Fig. 5E, the average EMG signals from Fig. 2 are replotted, with the reconstructed EMG based on the synergy analysis superimposed. There is a good resemblance between the two signals, indicated by their small differences for the different muscles (Fig. 5E).

To examine differences in the muscular coordination patterns over IDs, we examined how the *W*_*basis*_ were employed. The results suggest a difference in recruitment of *W*_*basis*_ per ID (Fig. 5C), for instance *W*_*basis*_ 2 and *W*_*basis*_ 7 appear to be most selected when ID is high or low, whereas selection of *W*_*basis*_ 3 shows the inverse and *W*_*basis*_ 5 is used increasingly more with higher IDs. Additionally, *PCA*_*syn*_ analyses on the temporal activation profiles *H* (Fig. 5B) showed that the score of the first component explained most of the variance between IDs: 65.4%, 62.6%, 72.7%, 83.0%, and 79.1%, for increasing IDs respectively. Scores of *PCA*_*syn*_ components two to seven explained less than 30% of variance and were similar per ID (see Fig. 5D for *PCA*_*syn*_ scores of the first and second components). This first component gradually varied with higher IDs, especially around the target approach (40–50% and 90–100% of cycle). A correlation of scores of the first *PCA*_*syn*_ components was calculated to provide us with a measure of similarity between temporal activations between IDs. We applied a bootstrapping analysis with 1000 samples per pair of IDs to assess the robustness of the correlation. The analysis showed that the scores for ID 3.3, ID 4.2 and ID 5.1 were significantly correlated (all p < 0.001) and the scores for ID 5.1, ID 6.0 and ID 6.9 were significantly correlated (all p < 0.001). Scores of ID 3.3 and ID 4.2 were not significantly correlated to those of ID 6.0 and ID 6.9 (all p > 0.05, average p = 0.55). Pearson’s correlations of scores of the second *PCA*_*syn*_ components were all significant (all p < 0.001), indicating its lack in explaining any difference between IDs. Note that again no information about group-level statistics can be assessed here due to our methodology (see above).

## Discussion

The present study aimed to explore the dynamical organisation existing between behavioural and muscular levels in Fitts’ task. More specifically, it aimed to determine whether, when the behaviour switches from one dynamical regime (limit-cycle) to another (fixed point), coordination of muscular activations also displays specific signatures of a dynamical phase transition, i.e. a reorganisation of muscular coordination and a loss of stability of muscular coordination at the vicinity of the behavioural phase transition. We directed changes in behavioural regime by manipulating accuracy constraints and tracked the concomitant muscular activations of 12 arm and shoulder muscles by surface EMG. We devised methods to analyse the dynamical coordination and variability of the muscular patterns, based on which we showed two necessary conditions of a phase transition at muscular level. To our knowledge, in spite of a number of debates about “whether muscles matter in phase transitions” during the last decade (for instance see^10^,^11^), it is the first (successful) attempt demonstrate that the muscular system follows the general principles of pattern formation in complex nonlinear systems.

As a prerequisite, we demonstrated the typical changes in the dynamical regime of behaviour when the target width decreased, thereby confirming target size acts as a control parameter of behavioural transition in Fitts’ task^4^,^5^,^6^,^9^. This was illustrated in the present study by the analysis of both phase flow trajectories and kinematics (see supplementary material). Taken together, the analyses indicated a transition from a limit cycle to a fixed point regime at ID ~4.9 and 5.14 bits for kinematics and phase flow respectively (see Fig. 1B). These results extend previous findings in Fitts’ task to a multijoint movement of the upper-limb performed in the anterior-posterior direction.

Matching this transition in behaviour with the analyses carried out on EMG data (Functional Connectivity Dynamics (FCD) and muscle synergies), we found two typical signatures accompanying a phase transition.

First, the phase transition in behaviour was accompanied, at the muscular level, by a peak in variability of coordination patterns. This conclusion was supported by the results of FCD analysis of muscle activations data. By tracking the overall changes in correlation between muscular activations during a trial, a proxy of variability was obtained^25^. Results showed an increased switching (related to FCD’s jump length) between functional correlations in parallel to the behavioural transition from limit cycle to fixed point regime (Fig. 3). After the behavioural transition, variability in correlational profiles was lower and the value at ID 6.9 was close to the value at ID 3.3. Thus, the general profile of functional connectivity dynamics observed over the different IDs reflected stability, loss of stability and re-stabilisation of muscular coordination before, during, and after the transition, respectively. This observation mimics critical fluctuations classically observed during a phase transition in numerous complex, nonlinear dynamical systems, including brain and behaviour^32^. These results might indicate that during a specific behavioural regimes (be it limit cycle or fixed point) a smaller set of the dynamical repertoire was used by the muscular system, while around the behavioural transition a larger part of this space was visited^25^. In other words, the ‘exploration’ was considerably lower during specific behavioural regimes than around the behavioural transition. This interpretation is consistent with the prediction that around the transition, the neuro-muscular system is in a metastable regime, which leads to random exploration of the different coordination states^20^.

Second, synergy analysis suggested that the altered variability of muscular activations captured by the FCD corresponded to a change in the temporal activations of muscular coordination patterns. Strikingly, the synergy analysis^33^,^34^,^35^,^36^ per 2-cycle bin suggested that a reorganisation of muscular coordination occurs by the assembly of seven similar muscle synergies for all IDs, adjusted in their temporal activation per ID (Fig. 4). This indicates that the movement in the forward-backward direction strongly constrains (or even over-determines) the nature and number of muscular patterns itself, while target size induces dynamical changes in their organisation (which is consistent with its status of control parameter at behavioural level, see^4^,^5^,^6^). This consistency of *W*_*basis*_ between conditions or with different task constraints has been shown in many papers, e.g. see the papers of d’Avella *et al*. on fast-reaching movements and reaching in different directions^30^,^31^). To examine differences in the nature of the synergy activation, we looked into differences in the quantity of activation per ID and differences in the temporal activation *H*.

Interestingly, the *PCA*_*syn*_ on *H* showed that IDs can be separated based on the score of the first *PCA*_*syn*_ only (Fig. 5D). As time-dependent activation profiles (*H*) for high IDs were not similar to the profiles for low IDs, it strongly suggests there is a distinction in temporal activation of the synergies between high and low IDs. This can be interpreted as a reorganisation of muscular coordination patterns, which occurs at ID 5.1 in parallel to the phase transition in dynamical regime seen in behaviour. These changes in time-dependent activation profiles during a transition in movement are comparable to what was recently found by Hagio *et al*.^27^ and by Ivanenko and colleagues^28^,^29^ who studied muscle synergies during gait transitions. These authors found the transition between walking and running not to be caused by different synergies (*W*_*basis*_), but by the regulation of the time-dependent activation profiles (*H*) of specific synergies. This strongly suggests that the production of transitions between two dynamical regimes behaviour require a reorganisation of muscular coordination patterns in the form of a change in the phasing of synergies. This change in the phasing would not be seen in the presence of a mere change in activation within a certain regime. A simple parametrisation of the variables would be represented by a simple adjustment in the amplitude and/or time duration of the temporal activation of the synergies. This would imply an increased activation or a longer activation of the synergy, respectively, without changing the timing (nature) of the activation. However, as we normalised our data in size and amplitude to enable comparisons, these simple modifications are not shown in our data. In contradiction, the change in phasing of the synergies–i.e. the changed shape of the temporal activation patterns *H*–that we find when comparing IDs implies that the synergies are differently used. This signifies the presence of a reorganisation in activation patterns.

The differences in muscular activations over IDs could also be explained by the fact that each synergy has a specific function and is, depending on ID, more or less frequently activated (Fig. 5C). For instance, *W*_*basis*_ 3, which denotes cocontraction between biceps and triceps, is increasingly more often used around the phase transition in behaviour. Such co-contraction, presumably corresponding to a freezing of the degrees of freedom, is usual for tasks where there is variable control^1^.*W*_*basis*_ 2 and *W*_*basis*_ 7 represent the main forward and backward movement of the upper limb, respectively. These synergies are most often used for high and low IDs, indicating that these principal movements were less routinely used during the transition between dynamical regimes. This could imply that the solution space of movements is explored more extensively, leading to less typical movements. Together, the changes in temporal activations *H* and the use of the synergies over IDs show predictable changes in how the repertoire of synergies is organised to comply to task demands. This interpretation extends the framework of coordination dynamics to the muscular level.

In summary, our results show three typical signatures of a phase transition at the muscular level: an increased variability towards the transition ID, lower variability after the behavioural transition, and a reorganisation of muscular coordination patterns corresponding to limit cycle and fixed point regimes, respectively. This set of convergent observations makes the existence of a phase transition at muscular level very likely. However, formally, since we grasped neither the order parameter nor the topology of the state space of the muscular system, we cannot categorically assert that such a phase transition indeed exists. We did show, though, that when behaviour shows a phase transition in Fitts task, muscular coordination reorganises accordingly. This is a first step and a strong indication that consistent changes in coordination processes exist across behavioural and muscular levels.

During the synergy extraction and selection (the NNMF procedure), we determine the number of synergies by taking the least number of synergies that account for at least 90% of the EMG signal. Therefore, here we discard 0% up to 10% of the information captured by the EMG signal, thus keeping 90–100% of the information. These synergies are then clustered per ID and averaged. The temporal activation patterns associated with these basic synergies then are subjected to the PCA analysis. The first component of this latter analysis explains 65.4%, 62.6%, 72.7%, 83.0%, and 79.1% of the temporal activation patterns with higher IDs, respectively. Including the second component would increase this to 94.3%, 92.2%, 87.9%, 91.6%, and 92.3%, respectively. Therefore, the PCA analysis gives a reliable representation of the original data given in the new Fig. 2. Importantly, by reducing the dimensionality of the data, the NNMF and PCA analysis reveal significant information about the muscular coordination that cannot be captured by classically analysing the original EMG data (Fig. 2). NNMF has been shown to be a very valuable method to obtain the muscular coordination patterns inherent to the ensemble of muscular activation signals. Combined with the PCA analysis, we gained a single variable that clearly shows a muscular reorganisation and that might indicate a phase transition if combined with other variables.

Whether this reorganisation of muscular patterns is abrupt or progressive can be debated. At first sight, some of our data (e.g. the existence of an intermediate point in jump length; Fig. 3) could be interpreted as indicative of a progressive reorganisation of muscular patterns, instead of a discrete transition. We contend, however, that this hypothesis is unlikely and can be due to our processing methods. We measured subjects’ performance at five specific IDs. The transition was always included in our range of IDs and the transition most often happened at the ID 5.1. However, some transition might have happened at higher or lower IDs (4.1 or 6.0 bits), thereby influencing, on average, our outcome measures. In support of this hypothesis, a comparable pattern of results was shown by Kelso and colleagues, who measured the phase difference between two fingers with increasing frequency in bimanual coordination^32^. Specifically, the existence of an intermediate point suggested some gradualness in the change of relative phase (see Fig. 2 in^32^). This point was not considered, however, by Kelso *et al*. (i.e., it was regarded as an artefact). Instead, they argued in favour of an abrupt phase transition at behavioural level (i.e., over a half cycle), which corresponded to the patterns empirically observed^37^. Thus, what appears to be a gradual change in our figures does not argue against the existence of discrete transitions.

It is interesting to note that the question of how the muscles are coordinated in the neuro-musculo-skeletal system has received only limited attention from the dynamical system approach (exceptions are^10^,^11^). Our findings suggest that the muscular sublevel reorganises as a complex dynamical system. Even if never conceptualised as such in the literature (to our best knowledge), this is not totally surprising as the muscular interface between brain and behaviour is inherently complex and non-linear. As an illustration, the nervous system has to deal with mastering numerous degrees of freedom to generate muscular coordination patterns (i.e., activation of a huge number of motor units, reduction of muscular redundancy). In addition, muscles are thixotropic^38^,^39^ and the relationship between muscle activation from the nervous system (measured by EMG) and joint kinematics (produced by the sum of all the muscle forces around the joint) holds curvilinear relationships with respect to fibre length, movement velocity, firing frequency of motor units, and moment arm of the tendon, e.g.^12^,^13^. It is unknown whether any pattern at the muscular level found in EMG will also be present in muscle force. As it is currently impossible to measure *in vivo* individual muscle forces (though see e.g.^16^ for advancements in estimations), it is still to be determined whether a dynamical organisation also exists at the level of muscle forces. As a dynamical coupling exists at the level of behaviour, one can expect that it should be also the case at the level of the coordination between forces.

In conclusion, in the present study, our general hypothesis was that the transition between behavioural dynamical regimes expresses consistent changes in coordination processes across the different levels of description (i.e. brain, behaviour, and muscular). By analysing the organisation of muscular coordination during a transition in behavioural dynamical regimes, we explored the dynamics at muscular level and its relation with the behavioural level.

Our results confirmed that, in Fitts’ task, target size may be considered as a control parameter that induces a destabilisation and a reorganisation of coordination patterns at different levels of the NMSS, including the muscular level. Indeed, according to our hypothesis, our data showed that when behaviour undergoes a phase transition to a different dynamical regime (corresponding to a different mode of movement control^4^), muscular coordination also displays some classical signatures of a phase transition (i.e., a reorganisation in coordination patterns and a concomitant increase and decrease in the variability of muscular activations around and beyond a critical ID, respectively). Importantly, the present study shows that the dynamical organisation of the muscular subsystem can be captured by analysing EMG data. These findings are striking due to the inherently complex and nonlinear relationship between muscular activation and behaviour. They suggest that muscular organisation is functionally linked to brain and behavioural organisation and follows general principles of pattern formation in complex, nonlinear systems^2^,^3^.

## Methods

### Experimental setup and design

Fourteen right-handed healthy volunteers (age mean ± std 23.2 ± 2.0 years, 8 male) completed the experiment. None of them suffered from known neurological or muscular disorders. Volunteers were asked to refrain from exercise and the consumption of alcohol 12 hours before participation (confirmed by questionnaire). All signed an informed consent form before the start of the experiment. The experimental protocol was approved by a local ethics committee of Aix-Marseille Université and was carried out in accordance with the Declaration of Helsinki.

Participants’ task was to slide a stylus in an anterior-posterior direction upon a graphical tablet between two targets of varying sizes. There were five conditions (*A* = 20 cm, ID equally spread between 3.3 to 6.9 bits) of 40 movement cycles (i.e., 80 aiming movements). Each condition was repeated four times. Repeats were blocked per ID and IDs were randomly ordered. This is similar to^4^, whereas an important difference is that an anterior-posterior movement was used to produce a multijoint motion to engage more muscles while realising the task.

Positional tablet data were sampled at 250 Hz. During each trial, surface EMG was recorded at 1925 Hz with a Trigno Wireless EMG system (Delsys Inc, USA) from the belly of the following twelve muscles (/parts): brachioradialis (BR), pronator teres (Pron), biceps brachii short head (BicSho), biceps brachii long head (BicLo), brachialis (Brach), triceps lateral head (TriLat), triceps long head (TriLo), pectoralis (Pect), deltoid anterior (DeltA), deltoid medialis (DeltM), deltoid posterior (DeltP), and teres major (TerM). Cohesive bandages (Lastopress 7 cm × 1.5 m, Hartmann Group, Germany) provided extra support for the electrodes. Before and after the experiment, participants executed maximal voluntary contractions of pushing or pulling a vertical pole in an anterior and posterior direction respectively (three repeats, lasting ~5 seconds each with 2 min rest in between) while verbally encouraged. Further analysis was carried out offline using custom written MATLAB scripts (Mathworks MATLAB 2012b, USA).

### Kinematical analyses

Positional data were resampled to 100 Hz, partitioned in cycles based on reversal points of the stylus position and the data of each trial’s first three and last two cycles were removed. For the remaining 70 half-cycles, we calculated the movement time (*MT*), the acceleration time (*AT*) and deceleration time (*DT*), which were pooled over participants. The effect of ID on *MT*, *AT*, and *DT* were analysed using a repeated measures ANOVA with ID as within-subject factor and α = 0.05. Where necessary, the Greenhouse-Geisser adjustment was applied to correct for violations of sphericity. Significant effects were subjected to a Student t-test with Bonferroni corrections to test for differences between IDs.

### Phase flow analyses

To verify the appearance of a limit cycle or fixed point regime per ID, vector field and phase flow analyses on the position and velocity time-series was reconstructed. These methods are widely used in the literature. The underlying concepts and methods of these analyses were described in detail by Van Mourik and colleagues^40^ and by Friedrich and Peinke^41^,^42^. A vector field reconstruction separates the deterministic components of the system dynamics from the random, or stochastic, components. The time evolution of a deterministic process of biological motion is often under the impact of random (i.e. stochastic) fluctuations. These components can be referred to as the diffusion and drift components, respectively. They can be separated via computation of its conditional probability matrix *P*(*x*, *y*, *t*|*x*_*o*_, *y*_*o*,_ *t*_*o*_), indicating the probability of the system to be at state (*x*, *y*) at time *t* when knowing the current state (*x*_*o*_,*y*_*o*_) at time step *t*_*o*_. To focus on the dynamics’ deterministic (i.e. the drift) component, we computed the conditional probability matrix of our position *x*(*t*) and velocity *y*(*t*) time-series over a 99 × 99 equally bin-sized grid for each trial. Per bin, the deterministic components were calculated as:


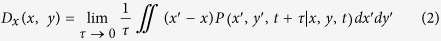


and


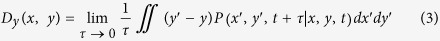


These deterministic components numerically specify the system’s vector field in the phase space. Therefore, the components reconstruct the flow that unambiguously determines the dynamical regime of the system. For each bin, we calculated the angle *θ* between neighbouring velocity vectors for the first two deterministic components (i.e., position and velocity). The maximum angle (*θ*_max_) was determined to test for the existence of fixed points (*θ*_max_ ~180°) or limit cycle (*θ*_max _≪ 180°). A sigmoid was fitted through the data points of *θ*_max_ as a function of ID according to


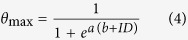


and the point of inflection identified the transition between the regimes.

### Functional Connectivity Dynamics

We used and adapted functional connectivity dynamics (FCD) analysis, which was previously utilised for fMRI data^25^, to study inter-trial variability in EMG data. First, the EMG data of each muscle separately was normalised in size according to its peak value recorded during the maximal voluntary contractions. The normalised EMG was cut per cycle based on positional data, band-pass filtered between 10 and 450 Hz (2^nd^ order Butterworth dual-pass filter), rectified and low-pass filtered at 5 Hz (4^th^ order Butterworth dual-pass filter). The EMG data per cycle were then resampled by interpolation to *N*_*p*/*c*_ = 100 time points. For every participant, ID, and repetition we correlated the EMG signals of all recorded muscles over a two cycle sliding window (e.g. 200 time points; Matlab function corr with option ‘coef’, full overlap between windows). Consequently, this resulted in 3300 symmetrical *N*_*m*_ × *N*_*m*_ (muscle-by-muscle) FC matrices, which each contain the correlations amongst all muscles at a specific time window. We subsequently correlated consecutive FC matrices to finally obtain a symmetrical *N*_*t*_ × *N*_*t*_(time-by-time, *N*_*t*_ = 3299) functional connectivity dynamics (FCD) matrix showing changes in correlations over the trial (see Fig. 3A for an example FCD). We then converted the FCD to a “jump length” matrix by subtracting the matrix from 1 (1 minus FCD). A jump length is the correlational distance of the functional connectivity dynamics between consecutive time windows. Jump length matrices were concatenated over the four repetitions.

To numerically quantify the amount of variability per ID, we calculated the median jump length size. We used the 204th diagonal of the jump length matrices, which is the first diagonal which does not include overlap between windows and thus does not comprise autocorrelation, and calculated the median jump length over this diagonal. This jump length distribution (*JLD*) captures the statistics of the muscular coordination patterns’ fluctuations, therefore representing a measure of the variability of those patterns. The effect of ID on the median *JLD* was tested with a repeated measures ANOVA with ID as within-subject factor and *α* = 0.05. We used the Greenhouse-Geisser adjustment whenever the assumption of sphericity was violated. Significant effects were subjected to a Student t-test with Bonferroni corrections to test for differences between IDs.

### Synergy extraction

To study any reorganisation in muscular activation patterns, for each participant and each ID separately, spatial synergies (*W*) and associated activation patterns over time (*H*) were extracted from the pre-processed EMG signal per bin of 2 movement cycles (i.e., *N*_*p*_ = 200 points) cf.^23^. Following DeMarchis and colleagues (2013), we applied the method per bins as we hypothesised that the number and selection of synergies might not be constant within a trial. For each matrix containing EMG from *N*_*m*_ = 12 muscles over 2 movement cycles, non-negative matrix factorisation (NNMF) was applied^43^. NNMF is an iterative optimisation method that minimises the normative error-matrix of


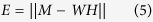


where *M* is the *N*_*m*_ × *N*_*p*_ matrix of recorded EMG signals, W is an N_m_ × N_s m_atrix containing the relative activations of each of the muscles with N_s_ being the number of synergies selected for extraction, and H is an *N*_*s*_ × *N*_*p*/*c*_ matrix containing the time-varying activity of each synergy. One to ten synergies were extracted for each of the *N*_*b*_ = 70 bins per ID and participant, and the NNMF was repeated 100 times for each value of *N*_*s*_ to ensure convergence to the minimum. The number of synergies chosen for further analysis was determined by calculating the variance accounted for (*VAF*) by the reconstruction *WxH* as:


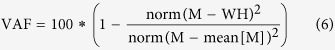


and by identifying the minimal number of *W* that could explain at least 90% of the variance of the EMG. *W* from all bins and participants were then normalised to a size of 1 and pooled per ID. Per ID, *W* were clustered by first performing a 2-dimensional Sammon’s mapping^44^ on the set of *W* of all bins and all subjects per ID and subsequently clustering the resulting Sammon’s map values (MATLAB function clusterdata with ward’s minimum variance method). In short, this procedure maps the set of *W* belonging to a *N*_*m*_-dimensional space to a set of 2-dimensional vectors while keeping the structure of the *W* intact. Using an error minimisation function, which contains a 2^nd^ order steepest descent procedure, the Euclidean distance between the *W* is preserved. This allows for a quantification of the number of clusters that underlie the set of synergy-vectors. Three to fifteen clusters were calculated for each ID. The minimum number of clusters where the correlation coefficient within each cluster had a median value above 0.5 was selected. *W* were then averaged within each cluster to calculate the representative synergies (*W*_*cluster*_) per ID. To consider changes in the use of *W*_*cluster*_ per ID, we adopted two approaches: 1) the number of times each *W*_*cluster*_ was extracted was identified per ID; 2) a principal component analysis (*PCA*_*syn*_) was applied on the concatenated matrix of *H* per ID. This gave us insight in the use of the synergy-repertoire and the variability in the amplitude of each *W*_*cluster*_ over time.

**Ethics.** The experiment was carried out in accordance with the Declaration of Helsinki and has been approved by a local ethics committee.

**Data Availability.** The article’s supporting data can be accessed online. MATLAB toolbox STABLE: http://math.bu.edu/people/mveillet/html/alphastablepub.html.

## Additional Information

**How to cite this article**: Vernooij, C. A. *et al*. Functional coordination of muscles underlying changes in behavioural dynamics. *Sci. Rep.* **6**, 27759; doi: 10.1038/srep27759 (2016).

## Supplementary Material

Supplementary Information
