# Supplementary material for: Functional coordination of muscles underlying changes in behavioural dynamics
Source: Sci Rep. 2016 Jun 10;6:27759. doi: 10.1038/srep27759 (PMC4901262; doi:10.1038/srep27759)

Functional coordination of muscles underlying changes in behavioural dynamics – SUPPLEMENTARY INFORMATION

Carlijn A. Vernooij^1*^, Guillaume Rao^1^, Dionysios Perdikis^2,3^, Raoul Huys^2^, Viktor K. Jirsa^2^, Jean-Jacques Temprado^1^

^1^Aix-Marseille Université, CNRS, Institut des Sciences du Mouvement UMR 7287, 13288, 13009, Marseille, France

^2^Aix Marseille Université, Inserm, Institut de Neurosciences des Systèmes UMR_S 1106, 13005, Marseille, France

^3^Max Planck Institute for Human Development, Center for Lifespan Psychology, Berlin, Germany

Corresponding author:

Carlijn Vernooij

Aix-Marseille Université

Faculté de Sciences du Sport & CNRS

Institut des Sciences du Mouvement E.J. Marey UMR7287

163 Avenue du Luminy, CP 910

13009 Marseille, France

Email: carlijn.vernooij@univ-amu.fr

**SUPPLEMENTARY MATERIAL – Kinematical results**

We were interested in the actual difficulty produced by the participants (effective ID; IDe) as a function of the predetermined difficulty (ID). Per half-cycle, the effective amplitude (Ae) and the effective target width (We) were calculated as the average distance between consecutive extremes and 1.96 times the standard deviations of the extremes, respectively, to determine the effective ID per trial as. IDe gradually increased for movements to smaller target sizes (mean ± standard error: 4.60±0.07, 5.23±0.09, 6.02±0.05, 6.69±0.07, 7.47±0.13; F_1.55,64.96_ = 235.27, η = .85, p < .001).

**Figure S1A** depicts the movement time (MT), acceleration time (AT), and deceleration time (DT) as a function of IDe. Although MT increases quite linear with higher IDe, this does not seem to be the case for AT and DT. To study this discontinuity, the AT/DT ratio was calculated for all pooled data points (280 data points in total), which was divided into two parts which size varied between 30 and 250 data points. For each separation, a linear regression was calculated for both parts. The separation where the sum of squared errors of the two parts was smallest denoted the breakpoint in AT/DT ratio. This breakpoint appeared at an IDe of 6.05 bits, or an ID of 5.14 bits.


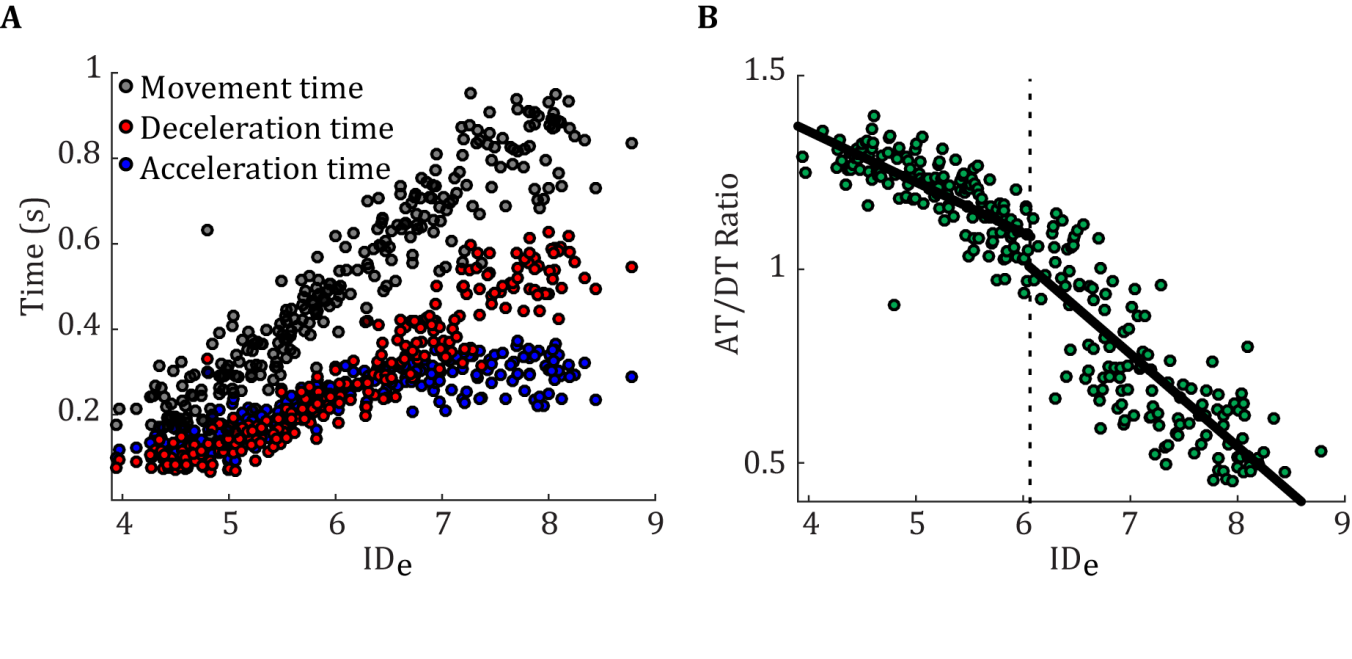


**Figure S1**

*(A)* Movement time and its partitioning of all participants. The horizontal axis represents IDe; the vertical axis time (s). Each dot is the average across repetitions per participant. Grey, red and blue dots represent MT, deceleration and acceleration phase, respectively. *(B)* The ratio between AT and DT as a function of IDe. A breakpoint in the ratio can be seen at an IDe of 6.05 bits.

**SUPPLEMENTARY MATERIAL – Glossary**


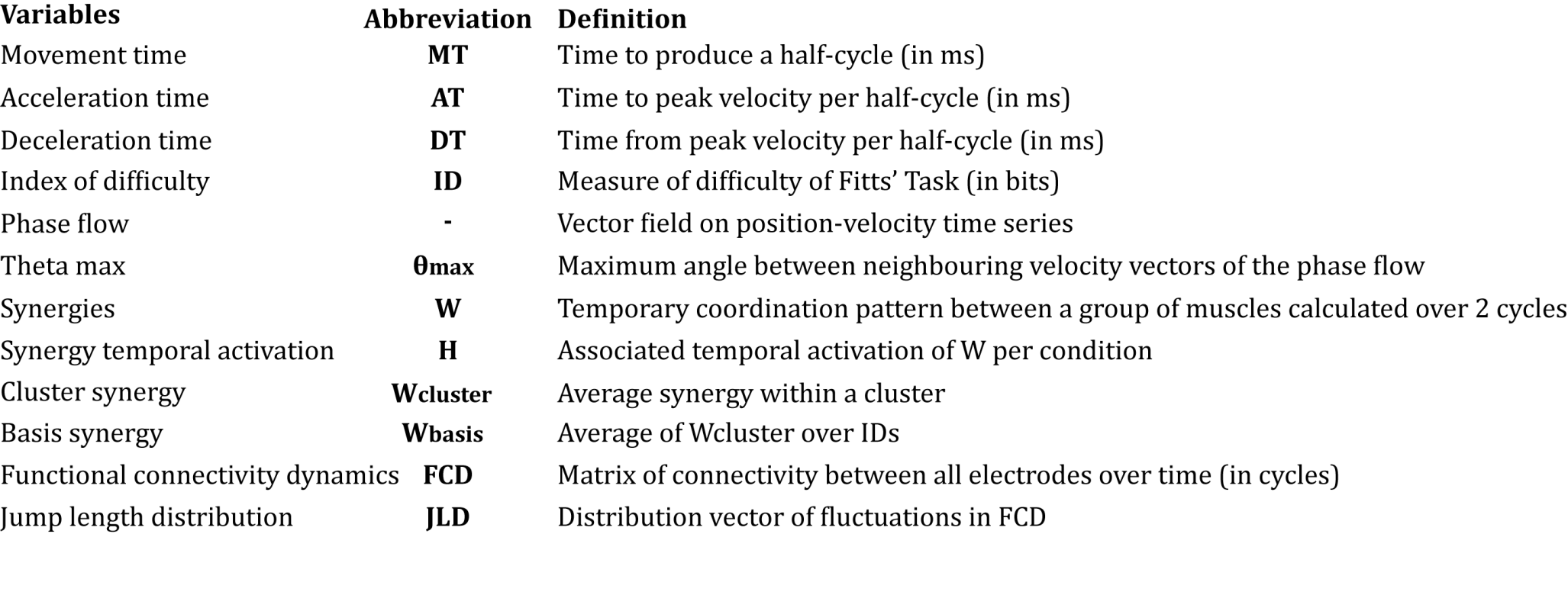


**SUPPLEMENTARY MATERIAL – Flow chart analyses**


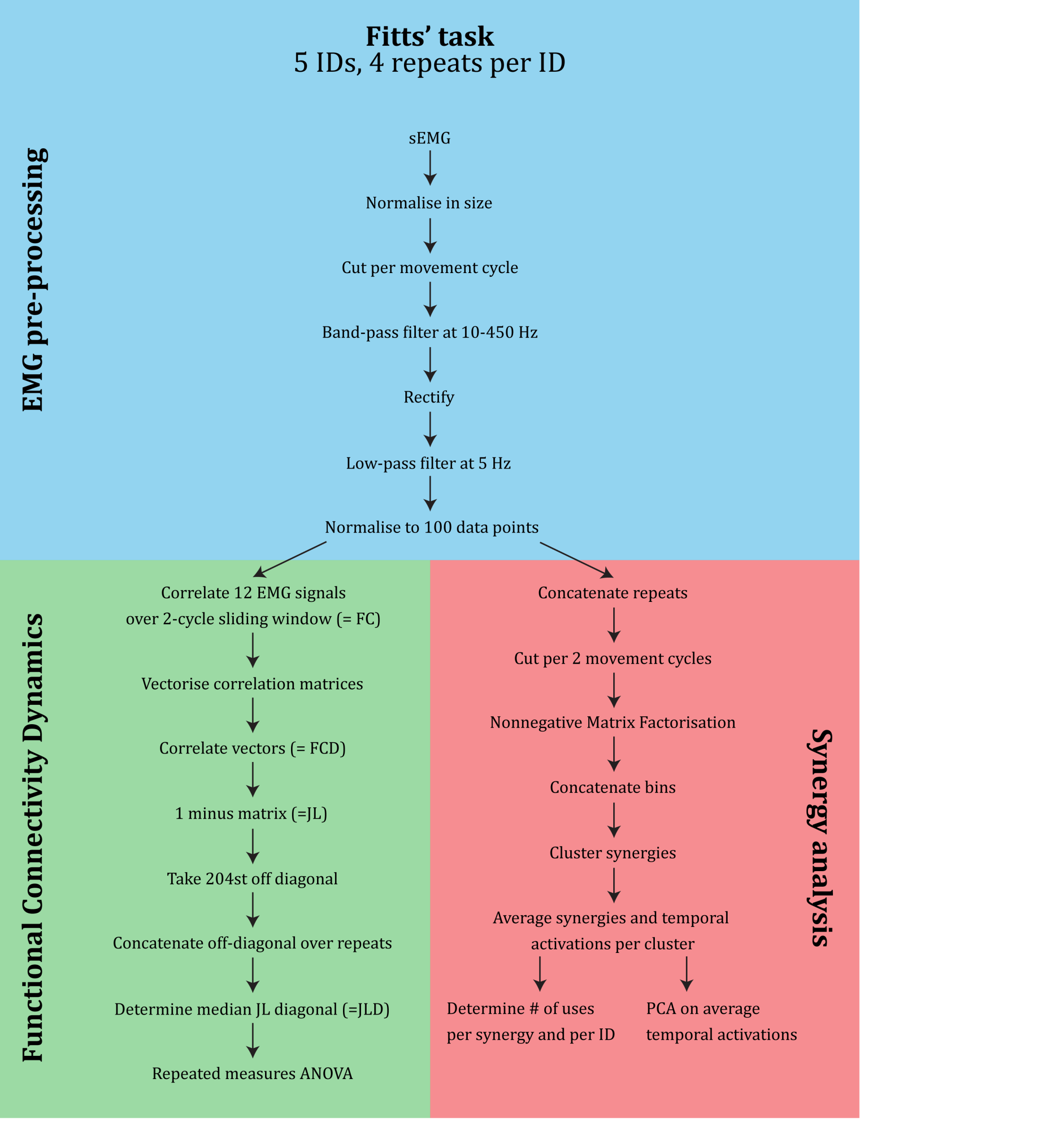

Supplement: Supplementary Information [file srep27759-s1.doc]
